# Bone Tissue Engineering in Rat Calvarial Defects Using Induced Bone-like Tissue by rhBMPs from Immature Muscular Tissues In Vitro

**DOI:** 10.3390/ijms23136927

**Published:** 2022-06-22

**Authors:** Tatsuhide Hayashi, Masaki Asakura, Mayu Kawase, Masakazu Matsubara, Yasuaki Uematsu, Akimichi Mieki, Tatsushi Kawai

**Affiliations:** Department of Dental Materials Science, Aichi Gakuin University School of Dentistry, 1-00 Kusumoto-cho, Chikusa-ku, Nagoya 464-8650, Japan; masaki@dpc.agu.ac.jp (M.A.); kawase-m@dpc.agu.ac.jp (M.K.); ag193d17@dpc.agu.ac.jp (M.M.); cookdo-tablet@docomo.ne.jp (Y.U.); akimichi.mieki@gmail.com (A.M.); kawaita@dpc.agu.ac.jp (T.K.)

**Keywords:** bone tissue engineering, immature muscular tissue, recombinant human bone morphogenetic proteins, induced bone-like tissue, expanded polytetrafluoroethylene

## Abstract

This study aimed to induce bone-like tissue from immature muscular tissue (IMT) in vitro using commercially available recombinant human bone morphogenetic protein (rhBMP)-2, rhBMP-4, and rhBMP-7, and then implanting this tissue into a calvarial defect in rats to assess healing. IMTs were extracted from 20-day-old Sprague-Dawley (SD) fetal rats, placed on expanded polytetrafluoroethylene (ePTFE) with 10 ng/μL each of rhBMP-2, BMP-4, and BMP-7, and cultured for two weeks. The specimens were implanted into calvarial defects in 3-week-old SD rats for up to three weeks. Relatively strong radiopacity was observed on micro-CT two weeks after culture, and bone-like tissue, comprising osteoblastic cells and osteoids, was partially observed by H&E staining. Calcium, phosphorus, and oxygen were detected in the extracellular matrix using an electron probe micro analyzer, and X-ray diffraction patterns and Fourier transform infrared spectroscopy spectra of the specimen were found to have typical apatite crystal peaks and spectra, respectively. Furthermore, partial strong radiopacity and ossification were confirmed one week after implantation, and a dominant novel bone was observed after two weeks in the defect site. Thus, rhBMP-2, BMP-4, and BMP-7 differentiated IMT into bone-like tissue in vitro, and this induced bone-like tissue has ossification potential and promotes the healing of calvarial defects. Our results suggest that IMT is an effective tissue source for bone tissue engineering.

## 1. Introduction

Bone is an active metabolic process that is important as a supportive and essential tissue for maintaining life processes. Bone fractures, defects, and tumors are typical bone diseases and require minimal intervention to recover fully. However, serious cases of bone diseases may not heal naturally. General clinical applications of bone fractures and defects include reconstructive surgery and bone transplantation [1]. The current procedures for acquiring bone for these surgeries include autografts, allografts, and xenografts. Autografts are still regarded as the gold standard, and both autografts and allografts are limited by donor tissue availability and donor site morbidity, while both allografts and xenografts have the risk of graft rejection and disease transmission [2].

Tissue engineering is a research field combining the principles of engineering and bioscience to achieve human tissue/organ regeneration or reconstruction. Tissue engineering aims to develop engineered tissues or substitutes created in vitro to restore, maintain, or improve tissue function [3,4,5]. For example, the bone tissue engineering strategy involves the interaction of cells/tissues, osteoinductive cytokines/growth factors, and biocompatible and osteoconductive scaffolds, which are recognized as potential ways to create biological tissue substitutes for regenerating bone defects [6,7].

Cells for tissue engineering must have the potential for high proliferation and differentiate into specific cell types. Therefore, stem cells, such as bone-marrow-derived mesenchymal stem cells (BMSCs), adipose-derived mesenchymal stem cells (ASCs), hematopoietic stem cells (HSCs), periosteum-derived stem cells (PSCs), dental pulp-derived stem cells (DPSCs), and induced pluripotent stem cells (iPSCs), are used as cell sources for tissue engineering because they are multipotent [8].

The number of novel scaffolds that can be differentiated into target cells/tissues in vivo has been investigated for decades. The role of scaffolds in tissue engineering is to provide a base on/in which cells can differentiate and proliferate in three dimensions. Generally, natural polymers, such as collagen [9], agarose [10], and alginate [11], inorganic/organic scaffolds, such as bioactive glass/gelatin [12], and synthetic polymers, such as poly(lactic acid), poly (glycolic acid), and their copolymer poly (lactic-co-glycolic acid) [13,14,15], are used as scaffolds. Natural polymers can be enzymatically degraded, whereas synthetic polymers are degraded by hydrolysis. Calcium phosphates, such as β-tricalcium phosphate (β-TCP) [16], hydroxyapatite (HA) [17], and octacalcium phosphate (OCP) [18], are preferred for bone tissue engineering because calcium phosphate has osteoconductive ability and better mechanical properties than polymer scaffold materials.

Cytokines/growth factors that induce differentiation of cells into target cells are also essential for tissue engineering. Bone morphogenetic proteins (BMPs) are bone-forming factors that can induce chondrogenesis and osteogenesis at ectopic and orthotopic sites [19,20]. Through their sequence homology with other BMPs, approximately 20 members of the BMP subgroup have been identified and delivered in multiple groups of structurally related proteins. For example, BMP-2 and BMP-4 are highly related, BMP-6, BMP-7, and BMP-8 form another subgroup, and growth and differentiation factor (GDF)-5, also termed cartilage-derived morphogenetic protein (CDMP)-1, and GDF-7, also termed CDMP-3, are similar to each other [21]. The amino acid sequence of human BMPs was isolated from the bone and produced recombinantly [22]. In vitro, BMPs were found to have potent effects on various cells implicated in cartilage and bone formation. For example, they induce proteoglycan synthesis in chondroblasts and stimulate alkaline phosphatase activity and type I collagen synthesis in osteoblasts [23]. While there are many BMPs in existence, there are many reports that BMP-2, BMP-4, and BMP-7 specifically induce osteogenic differentiation of stem cells and progenitor cells in vitro and induce endochondral bone formation and repair bone defects in various animal models in vivo [12,24,25]. Thus, these BMPs have therapeutic potential for bone repair and regeneration.

Our previous experiments attempted to induce cartilage and bone from immature muscular tissue (IMT) in vitro using BMPs (extracted from decalcified bovine cortical bones) and expanded polytetrafluoroethylene (ePTFE) as a scaffold. In these experiments, almost complete cartilage was successfully induced in vitro [26] and only bone-like tissue was induced in an in-vitro osteoinduction experiment; however, this induced bone-like tissue has ossification potential within two weeks after implantation [27]. Therefore, this experiment aimed to induce bone-like tissue from IMT in vitro using recombinant human BMP (rhBMP)-2, rhBMP-4, and rhBMP-7; second, to confirm that this induced tissue has ossification potential in vivo; and finally, to implant this tissue into a calvarial defect in rats and assess the resulting healing.

## 2. Results

### 2.1. Micro-CT and Histological Observations of Cultured IMT

IMTs treated with rhBMP-2, BMP-4, and BMP-7 and control (treated with sterilized 4 mM HCl containing 0.2% bovine serum albumin [BSA] only) were cultured for two weeks (thereafter, cultured IMTs treated with rhBMP-2, rhBMP-4, and rhBMP-7 are represented as rhBMP-2 sample, rhBMP-4 sample, and rhBMP-7 sample, respectively). Subsequently, micro-CT and histological observations were performed.

**Figure 1:**
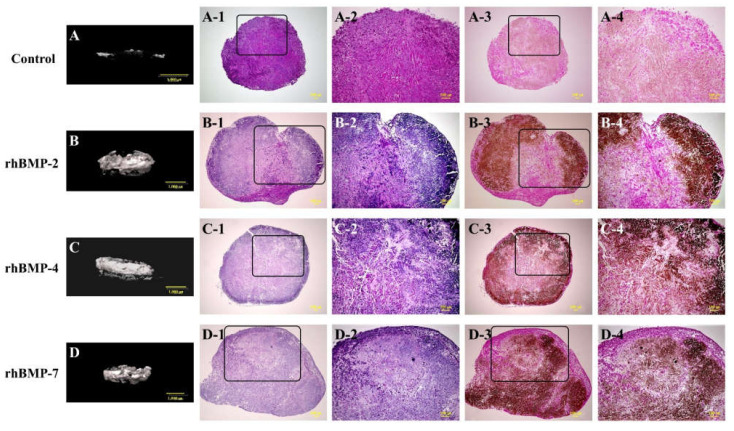
Typical micro-CT (**A**–**D**) and histological images of cultured immature muscular tissue (IMT), control (treated with 10 μL of sterilized 4 mM HCl containing 0.2% BSA only) and treated with 10 μL of each recombinant human bone morphogenetic protein (rhBMP)-2, rhBMP-4, and rhBMP-7. Samples were cultured for two weeks. A-1, -2, B-1, -2, C-1, -2, and D-1, -2 show hematoxylin and eosin (H&E) staining and A-3, -4, B-3, -4, C-3, -4, and D-3, -4 show von Kossa staining. A-2–D-2 and A-4–D-4 are magnified insert images of A-1–D-1 and A-3–D-3, respectively. Scale bar sizes in micro-CT and histological images are 1000 µm and 100 µm, respectively.

Representative micro-CT images and low- and high-magnification histological images are shown in Figure 1. In the micro-CT results, the control showed slight radiopacity. In contrast, relatively strong radiopacity was observed at the periphery of all rhBMP samples, and almost none were observed near the center. Histological observation showed that bone-like tissue, comprising osteoblastic cells and osteoids, was partially observed by hematoxylin and eosin (H&E) staining. Additionally, von Kossa staining showed strong mineral deposition, mainly in the periphery of the rhBMP samples. The results of von Kossa staining were consistent with those of the micro-CT. The control showed robust eosinophilic staining tissue by H&E staining and slight mineral deposition by von Kossa staining.

### 2.2. Electron Probe Microanalyzer (EPMA) Analysis

**Figure 2:**
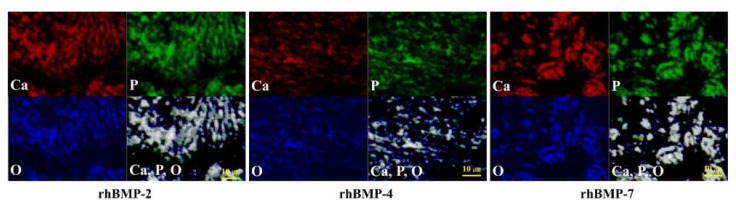
EPMA analysis of the elemental compositions of rhBMP-2, rhBMP-4, and rhBMP-7 samples two weeks after cultivation. Calcium (Ca; red), phosphorus (P; green), and oxygen (O; blue) were detected in the extracellular matrix. All three elements were confirmed to be at almost identical positions (white). Scale bar size is 10 µm.

The elemental compositions of the rhBMP-2, rhBMP-4, and rhBMP-7 samples two weeks after cultivation were examined using EPMA (Figure 2).

In each rhBMP sample, Ca, P, and O were widely detected in the extracellular matrix (ECM) and were confirmed to be at almost the same position based on synchronized image observations.

### 2.3. Surface Chemistry Analyses

**Figure 3:**
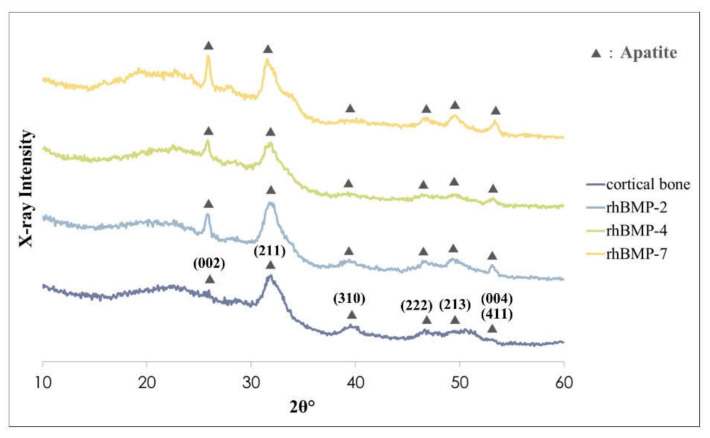
XRD patterns of rhBMP-2, rhBMP-4, and rhBMP-7 samples two weeks after cultivation and rat calvarial cortical bone. Black triangles show typical apatite peaks and their crystal planes were indexed according to JCPDS card 9-432.

**Figure 4:**
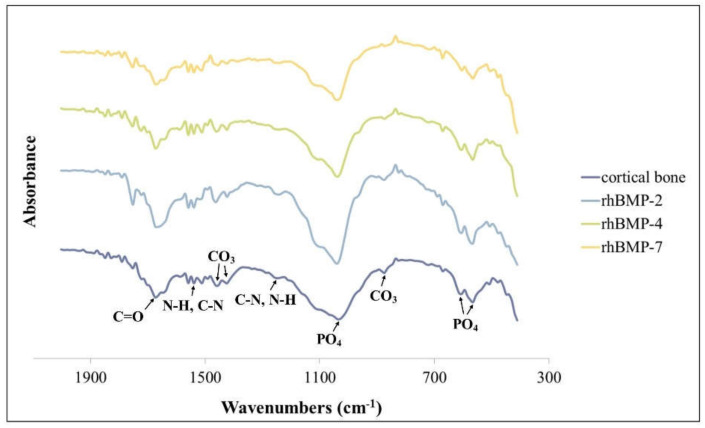
FT-IR spectra of rhBMP-2, rhBMP-4, and rhBMP-7 samples two weeks after cultivation and rat calvarial cortical bone.

The surface chemistry of each two-week cultured rhBMP-2, rhBMP-4, and rhBMP-7 sample was evaluated using X-ray diffractometry (XRD) and Fourier transform infrared (FT-IR) (Figure 3 and Figure 4).

The crystalline phases of all rhBMP samples showed similar patterns to those of the rat calvarial cortical bone. Typical apatite, such as hydroxylapatite and carbonate apatite peaks were confirmed in all rhBMP samples, and some apatite crystallization of rhBMP samples was higher than that of rat cortical bone. The broad peak at approximately 32° is the most typical apatite peak [28]. Crystalline planes of typical apatite peaks were indexed according to the Joint Committee on Powder Diffraction Standard (JCPDS) card 9-432 (Figure 3).

The FT-IR spectra of all rhBMP samples and rat calvarial bone showed absorption bands of characteristic proteins and minerals. The spectra of all rhBMP samples were similar to those of cortical bone. The proteins were primarily detected by absorption spectra around 1655 cm^−1^ (amide I; C=O), 1550 cm^−1^ (amide II; N-H and C-N), and 1250 cm^−1^ (amide III; C-N and N-H) [29]. The mineral phase was detected in the PO_4_ spectra around 1030 cm^−1^ and 600–560 cm^−1^ (characteristic crystalline phosphate phases) and in the C-O bands of CO_3_ around 1480, 1420, and 880 cm^−1^ (Figure 4) [30].

### 2.4. Micro-CT and Histological Observation of Subcutaneously Implanted Cultured IMT

**Figure 5:**
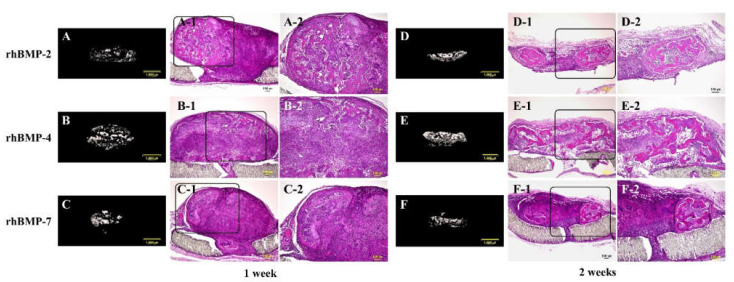
Typical micro-CT (**A**–**C** and **D**–**F**) and histological images of each subcutaneously implanted rhBMP-2, rhBMP-4, and rhBMP-7 sample into the backs of rats at one and two weeks. A-2, B-2, and C-2 are magnified insert images of A-1, B-1, and C-1, and D-2, E-2, and F-2 are magnified insert images of D-1, E-1, and F-1, respectively. Scale bar sizes in micro-CT and histological images are 1000 µm and 100 µm, respectively.

After one and two weeks of subcutaneous implantation of rhBMP-2, rhBMP-4, and rhBMP-7 samples, micro-CT and histological observations were performed (Figure 5). 

In the micro-CT results, strong radiopacity was granularly observed one week after implantation of all rhBMP samples, and these granular radiopacities were observed to become lumped together two weeks after implantation. Partial ossification was confirmed one week after implantation of all rhBMP samples by histological observation. Ossification was still partial, but ossification areas became wider and the bone matrix was more clearly observed in implanted rhBMP-2 and rhBMP-7 samples, particularly in the implanted rhBMP-4 sample, where the ossification area was identified almost throughout the tissue.

### 2.5. Micro-CT and Histological Observation of Cultured IMT Implanted in Calvarial Defect

**Figure 6:**
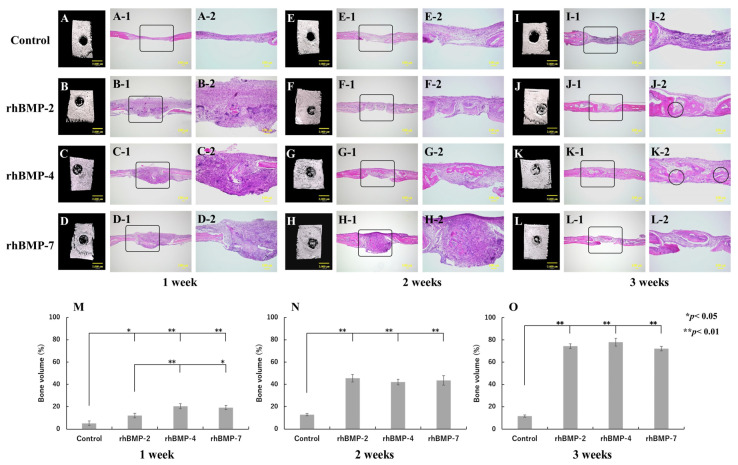
Typical micro-CT (**A**–**D**, **E**–**H**, and **I**–**L**) and histological images of each implanted rhBMP-2, rhBMP-4, and rhBMP-7 sample into the calvarial defects of rats at one, two, and three weeks. (**A**,**E**,**I**) are the control. A-2–D-2, E-2–H-2, and I-2–L-2 are magnified insert images in A-1–D-1, E-1–H-1, and I-1–L-1, respectively. The circles in J-2 and K-2 indicate the areas where the induced new bone integrated with the original calvarial bone. Comparison of the new bone volume of defect area (%) at one (**M**), two (**N**), and three (**O**) weeks after implantation. The data are shown as mean ± SD (*n* = 3). Scale bar sizes in micro-CT and histological images are 2000 µm and 100 µm, respectively.

rhBMP-2, rhBMP-4, and rhBMP-7 samples were implanted into rat calvarial defects for up to three weeks. Micro-CT and histological observations were carried out, and the volume of induced new bone was measured (Figure 6).

The control group did not show any radiopacity in the micro-CT images at the defect site and hardly showed new bone; however, fibrous tissue was mainly observed during histological observation in all experimental periods. In contrast, strong radiopacity was observed after one week in all rhBMP samples, although this was still partial. Two weeks after implantation, the radiopacity area became wider and the area was much wider at three weeks in the defect site. Histological observation confirmed slight ossification one week after implantation and dominant-induced new bone was observed after two weeks in the defect site. Furthermore, especially in the implanted rhBMP-2 and rhBMP-4 samples, the induced new bone seemed to integrate with the original calvarial bone after three weeks (Figure 6J-2,K-2).

The quantitative new bone volumes showed significant differences from one week after implantation between the control and each implanted rhBMP-2, rhBMP-4, and rhBMP-7 sample. In addition, comparing experimental groups, significant differences were observed between rhBMP-2 and rhBMP-4 samples and between rhBMP-2 and rhBMP-7 samples at one week after implantation but not after two weeks of implantation (Figure 6M–O).

## 3. Discussion

The bone formation process is complex and meticulously regulated, and bone is highly vascularized, with most blood vessels located within 100 μm of osteoblasts synthesizing and mineralizing the bone matrix [31]. Critical-sized bone defects are a challenging problem in medicine and dentistry, even in highly vascularized tissues, such as bone. Therefore, recent research has focused on tissue engineering strategies. The goal of tissue engineering is to provide living constructs that can integrate with the surrounding tissue. In the present study, we reported the use of IMT, which was cultured with rhBMP-2, rhBMP-4, and rhBMP-7 as cytokines, and ePTFE as a scaffold to induce bone-like tissue in vitro, and showed the status of rat calvarial defect healing after implantation of the rhBMP samples.

Multipotent stem cells have been isolated from various tissue types, such as the bone marrow, adipose tissue, umbilical cord, and dental pulp, and are used for bone regeneration and bone tissue engineering. BMSCs are among the most commonly used stem cells in bone tissue engineering because they are abundant, relatively easy to harvest and culture, and readily maintain their differentiation potential. Moreover, BMSCs readily differentiate into osteoblastic cells and possess greater osteogenic potential than chondrogenic or adipogenic potential [32]. ASCs are isolated from adipose tissue and are abundant and easy to harvest. ASCs can differentiate into osteogenic, adipogenic, myogenic, and neurogenic lineages, similar to BMSCs. In addition to their multi-lineage differentiation potential, ASCs have vasculogenic potential [32,33]. BMSCs and ASCs have a limited differentiation capacity, but iPSCs, derived and reprogrammed directly from adult somatic cells, could give rise to every type of cell in the body and propagate indefinitely. iPSCs hold enormous potential in the entire field of regenerative medicine, as they possess pluripotency and differentiation potential comparable to that of embryonic stem cells [34,35]. DPSCs, which have recently attracted particular attention in dentistry, are isolated from extracted third molar teeth through the enzymatic breakdown of DPSCs and are similar to BMSCs at various points, including their cell surface phenotype [36]. In addition to these, several stem cell types have been used in regenerative medicine and tissue engineering. IMT, mainly comprising myoblasts and myotubes, has been used in previous and current experiments instead of a stem cell source. The cell differentiation state in IMT corresponds to an intermediate stage between stem and mature cells, as determined by the expression of the primary myogenic regulatory factors, Myf5 and MyoD, and the secondary myogenic regulatory factors, myogenin and MRF4 [27]. IMT may not have pluripotency like stem cells; however, cartilage and bone-like tissue induction have been achieved in vitro using IMT and BMPs, and furthermore, this induced bone-like tissue has ossification potential in vivo [26,27]. In addition to IMT, progenitor cells, such as trabecular bone-derived progenitor cells and primary chondrocytes, have been used for bone tissue engineering [37,38].

BMPs are multifunctional cytokines that are members of the transforming growth factor-beta (TGF-β) superfamily. Currently, approximately 20 BMP family members have been identified and characterized [39]. All BMPs are secreted as precursor proteins with a hydrophobic stretch of 50–100 amino acids [40]. BMPs induce the formation of both cartilage and bone and play a role in several non-osteogenic developmental processes, including the regulation of cell proliferation, survival, differentiation, and apoptosis [41,42]. BMP-2, BMP-4, and BMP-7, in particular, are well-known potent osteoinductive cytokines that induce the osteogenic differentiation of pluripotent mesenchymal cell lines and promote the maturation of osteoblastic progenitor cells [43,44]. Moreover, there are many reports of successfully regenerated calvarial, femur, and tibia defect models of mammals using these BMPs [9,12,25,45,46,47,48]. In addition to those, it was reported that gene expression of BMP-2, BMP-4, and BMP-7 was significantly upregulated during fracture healing in a rat tibia fracture model [49], and BMPs reportedly suppress the expression of muscle-related genes, including myogenin and certain muscle kinases, and stimulate the expression of osteoblastic marker genes in the myogenic C2C12 cell line [50,51].

In cell differentiation experiments, the culture medium composition and supplement type introduced to the medium are also important. A well-known study described the differentiation of bone marrow cells into osteoblasts [52], and 10^−8^ M dexamethasone was used as a key supplement. As mentioned in the Section 4, the culture medium was prepared according to the method described in the aforementioned study, except that rhBMPs were used instead of dexamethasone. Furthermore, 10 mM CaCl_2_ was added to the culture medium in the present experiment because our most recent experiment showed that adding 10 mM CaCl_2_ in the culture medium promoted both the in-vitro calcification and ossification potential of induced bone-like tissue [53]. As a result of the two-week cultivation of IMT with rhBMP-2, rhBMP-4, and rhBMP-7 in this culture medium composition (α-MEM supplemented with 15% FBS, 50 μg/mL ascorbic acid 2-phosphate, 10 mM Na-β-glycerophosphate, 10 mM CaCl_2_, penicillin, and streptomycin), osteoblastic cells, osteoids, and strong calcification were histologically observed in each rhBMP sample. In EPMA analysis, Ca, P, and O were widely detected in the ECM at almost identical positions, indicating that the induced tissue was calcium phosphate tissue. In the XRD analysis, all rhBMP samples showed typical apatite crystalline peaks similar to those of rat cortical bone. Furthermore, the FT-IR spectra of all rhBMP samples showed amide groups (amide I, II, and III), which indicate protein–peptide bond, typical crystalline phosphate phase, and carbonate group spectra, which are similar to those of cortical bone, as well as the XRD results [29,30]. Although complete bone was not induced in vitro, probably because there was no nutrition supply from blood vessels, based on the evidence and these results, it is suggested that IMT has osteogenic differentiation potential. The differentiation of the IMT into bone-like tissue in vitro was confirmed by histological observations, elemental analysis, and surface characterization.

After two weeks of culture, each rhBMP sample was implanted subcutaneously into the backs of rats to confirm the ossification potential in vivo for up to two weeks. All implanted rhBMP samples showed strong radiopacity in micro-CT images from one week after implantation, and osteocytes and bone matrix were histologically observed one week after implantation. In addition, two weeks after implantation, robust osseous tissue was observed, particularly in the rhBMP-4 sample. These results indicate that the induced bone-like tissue has ossification potential in vivo.

The function of the scaffold in bone tissue engineering is to provide a three-dimensional framework for cells to attach, grow, and differentiate. Several scaffold components are required for successful incorporation and functionality, such as biocompatibility, biodegradability, adequate surface properties and porosities, osteoconductive and osteoinductive properties, recruitment of osteoprogenitor cells to defect regions, and controlled release of differentiation signals [54]. In general, as the scaffolds used for tissue engineering will eventually be implanted in the human body, the scaffold materials should be non-antigenic, non-carcinogenic, non-toxic, non-teratogenic, and process high cell/tissue biocompatibility, so that they do not trigger pathological reactions. Furthermore, the scaffold should biodegrade in the body after implantation. In our experiments, ePTFE, a non-biodegradable membrane material, was used as a scaffold at all times. Therefore, it will probably be removed from the cultured IMT prior to implantation into bone defects or bone augmentation; however, it is easy to remove ePTFE from cultured IMT because it is easy to exfoliate. Specifically, the main purpose of using ePTFE as a scaffold in this experiment was to provide a stable environment where the IMT could differentiate and grow into bone (bone-like tissue) rather than guide the IMT’s three-dimensional formation. Indeed, the cultured IMT showed a spherical form with an approximately 1.5 mm diameter; that is, the cultured IMT itself already has a three-dimensional shape [27]. Thus, it is unnecessary to be concerned about antigenicity, carcinogenicity, and toxicity after ePTFE implantation because it will be removed prior to implantation. In fact, all rhBMP samples were implanted into rat calvarial defects for up to three weeks after removing ePTFE.

The control (without rhBMP sample implantation) showed no radiopacity in the micro-CT images and hardly showed new bone histologically at the defect site in all experimental periods. In contrast, strong radiopacity was partially observed and ossification was histologically confirmed from one week after rhBMP sample implantation. Furthermore, particularly in the implanted rhBMP-2 and rhBMP-4 samples, new bone integrated with the original calvarial bone was observed three weeks after implantation. The new bone volume quantification showed significant differences between the control and rhBMP samples in all experimental periods. There was a statistically significant difference among experimental groups only after one week of implantation; however, since the bone defect healed well over time in the case of each rhBMP sample implantation, it is suggested that there are few differences in bone regeneration ability among rhBMP samples.

However, IMTs are extracted from fetal rats. We recognize that it is impractical to use fetuses for clinical application in humans. Therefore, we are currently attempting similar experiments using mature muscular tissue and have already obtained some valid results.

## 4. Materials and Methods

### 4.1. Recombinant Human BMPs

Commercially available rhBMP-2, rhBMP-4, and rhBMP-7 were purchased from R&D Systems (Minneapolis, MN, USA) and dissolved in sterilized 4 mM HCl (Kanto Chemical Co., Inc., Tokyo, Japan) containing 0.2% BSA (Invitrogen, Carlsbad, CA, USA) before use.

### 4.2. ePTFE Properties

ePTFE (GORE-TEX^®^; Japan Gore Inc., Okayama, Japan), with a thickness of 400 µm, was used as the scaffold. ePTFE is a non-biodegradable membrane derived from PTFE following a specially expanded processing procedure. ePTFE possesses many unique properties and functions, including biocompatibility, chemical stability, anti-thrombogenicity, weather and fire resistance, gas permeability, water repellency, non-water absorption, and toughness [26]. Prior to experimental use, ePTFE was cut into 5 × 5 mm sections and notched in the central part (approximately 2 mm across) to facilitate greater tissue anchorage to the membrane and sterilized with ethylene oxide gas.

### 4.3. IMT Isolation and Culture

IMT samples were removed from the forelimbs of Sprague-Dawley (SD) fetal rats at 20 days of pregnancy, according to a previously established protocol [26,27,53].

Briefly, pregnant SD rats were sacrificed using diethyl ether (Yoneyama Yakuhin Kogyo, Co., Ltd., Osaka, Japan) and the abdomen was disinfected with 70% ethanol (Ueno Chemical Industries Ltd., Osaka, Japan). Next, fetal rats were removed from the uterus through a celiotomy incision and washed carefully with sterilized ultrapure water. After the epidermis had flayed, the IMT was excised from the right and left forelimbs using forceps. IMT samples were approximately 2 mm^3^ in size with a wet weight of approximately 20 mg. The excised tissues were placed into a disposable tube containing 450 μL of medium and 10 μL (10 ng/μL) of rhBMP-2, rhBMP-4, and rhBMP-7. The tissue was homogenized using a pestle and incubated for 24 h at 37 °C in air containing 5% CO_2_ at 95% humidity in an incubator. The culture medium consisted of α-MEM (Life Technologies, Grand Island, NY, USA) supplemented with 15% fetal bovine serum (FBS; Life Technologies, Grand Island, NY, USA), 50 μg/mL ascorbic acid 2-phosphate (Sigma-Aldrich, St. Louis, MO, USA), 10 mM Na-β-glycerophosphate (Sigma-Aldrich, St. Louis, MO, USA), 10 mM CaCl_2_ (Kanto Chemical Co., Inc., Tokyo, Japan), penicillin (100 units/mL; Life Technologies, Grand Island, NY, USA), and streptomycin (100 μg/mL; Life Technologies, Grand Island, NY, USA). After 24 h of incubation, samples were placed on notched ePTFE, after which 10 μL of 10 ng/μL each of rhBMP-2, rhBMP-4, and rhBMP-7 was directly dropped onto the tissue, and 900 μL of culture medium was added to the culture plate. The control sample was treated with 10 μL of sterilized 4 mM HCl containing 0.2% BSA. The samples were cultured for two weeks at 37 °C. The culture medium was changed every other day, and rhBMP-2, rhBMP-4, and rhBMP-7 or sterilized 4 mM HCl containing 0.2% BSA were directly dropped onto the tissue throughout the first week of culture.

### 4.4. Micro-CT and Histological Observation of Cultured IMT

The IMT samples were observed two weeks after cultivation using micro-CT (R_mCT; Rigaku, Tokyo, Japan). The micro-CT was operated for 2 min at an acceleration potential of 90 kV with a beam current of 150 μA, ten-fold magnification, and cubic voxel size of 20 × 20 × 20 μm^3^.

Cultured IMT samples were fixed in 10% neutral formalin for five days. Fixed tissues were embedded in paraffin, and serial sections (4 μm) were obtained and stained with H&E and von Kossa according to standard procedures. Briefly, for von Kossa staining, samples were soaked in 5% silver nitrate (AgNO_3_; Kanto Chemical Co., Inc., Tokyo, Japan) after deparaffinization and then exposed to UV light for 1 h at room temperature. Samples were then carefully rinsed thrice with distilled water and treated with 5% sodium thiosulfate (Kanto Chemical Co., Inc., Tokyo, Japan) for 3 min to remove the background stain. Finally, the samples were counterstained with nuclear fast red (FUJIFILM Wako Pure Chemical Corp., Osaka, Japan) for 5 min and rinsed with tap water for 3 min.

### 4.5. Elemental Composition Analysis

EPMA (JXA-8530F; JEOL Ltd., Tokyo, Japan) analysis of the cultured IMT was performed to detect elemental levels of calcium (Ca), phosphorus (P), and oxygen (O). Before the analysis, the specimens were soaked in methyl methacrylate (FUJIFILM Wako Pure Chemical Corp., Osaka, Japan) and polymerized in an incubator (37 °C). Subsequently, the specimens were polished with a sequence of abrasives down to a 0.05-μm alumina suspension and cleaned in an ultrasonic cleaner. A thin carbon layer was vaporized on the surface to make it conductive. The probe was operated at an accelerating voltage of 15 kV and probe current of 50 nA, focused as an electron beam with a diameter of 50 μm on the specimen surface.

### 4.6. Surface Characterization

The crystalline phases of the cultured IMT were characterized using XRD (Ultima IV; Rigaku, Tokyo, Japan) at 40 kV and 40 mA. Scans were conducted over a 2*θ* angular range of 10° to 60° at 0.5°/min. Crystalline planes of typical apatite peaks were indexed according to JCPDS card 9-432. Additionally, FT-IR spectra of the cultured IMT were obtained using a FT-IR spectrometer (Diamond-20; JEOL, Tokyo, Japan). In this test, 128 scans were averaged to obtain a single spectrum. Moreover, the crystalline phases of rat calvarial cortical bone were determined by XRD and FT-IR for comparison.

### 4.7. Subcutaneous Implantation of Cultured IMT In Vivo

After two weeks of culture, IMT samples were carefully washed thrice with 1 M sterilized PBS prior to subcutaneous implantation. Three-week-old male SD rats were anesthetized with an intraperitoneal injection of 1 μL/g pentobarbital sodium (Somnopenty^®^; Merck & Co., Inc., Rahway, NJ, USA). After disinfection of the surgical site with 70% ethanol (Ueno Chemical Industries Ltd., Osaka, Japan) and shaving the fur, the skin on the back was dissected to approximately 20 mm. Cultured IMT samples were implanted subcutaneously into the backs of rats for up to two weeks to confirm the ossification potential in vivo.

### 4.8. Cultured IMT Implantation into the Calvarial Defect

After culturing for two weeks, the IMTs were carefully washed thrice with 1 M sterilized PBS. Three-week-old male SD rats were anesthetized with an intraperitoneal injection of 1 μL/g pentobarbital sodium (Somnopenty^®^; Merck & Co., Inc., Rahway, NJ, USA). After surgical site disinfection with 70% ethanol (Ueno Chemical Industries Ltd., Osaka, Japan) and shaving of the fur, the skin on the head of the rats was dissected to approximately 15 mm. The calvarial periosteum was then separated to expose the skull bone. After the flap was raised, a critical-sized bone defect (approximately 2 mm in diameter) was created in the skull’s left and right areas using a 1.5-mm diameter round-type burr under running water. Subsequently, the ePTFE was removed from the cultured IMTs, and samples were implanted into the calvarial defect. Untreated calvarial defects were used as controls. Finally, the skin was closed using a surgical stapler, and implantation was allowed to proceed for up to three weeks.

### 4.9. In Vivo Micro-CT and Histological Observation

The rats were sacrificed 1, 2, or 3 weeks after implantation, and the defect area was removed. The samples were then examined using micro-CT (R_mCT; Rigaku, Tokyo, Japan) with similar imaging conditions as those described in the previous sections. A region of interest (ROI) with a diameter of 0.5 mm encompassing the original bone defect was chosen. The volume of new bone in the ROI was measured (*n* = 3) using TRI/3D BON software (RATOC System Engineering Co., Ltd., Tokyo, Japan). The new bone volume was expressed as a percentage of the total bone volume per ROI, based on the defect size. After obtaining micro-CT images, the samples were fixed in 10% neutral formalin (FUJIFILM Wako Pure Chemical Corp., Osaka, Japan) for one week and then decalcified using 10% ethylenediaminetetraacetic acid (EDTA; Yoneyama Yakuhin Kogyo Co., Ltd., Osaka, Japan) for two weeks. Thereafter, histological observation by H&E staining was carried out as per the method described above.

### 4.10. Statistical Analysis

All values are expressed as the mean ± standard deviation. Statistical analyses were performed using one-factor analysis of variance, followed by Tukey’s *post hoc* test. *p* values of less than 0.05 and 0.01 were considered statistically significant. Values are expressed as the mean ± standard deviation (M ± SD).

## 5. Conclusions

Successful tissue engineering relies on a combination of cells/tissues, cytokines/growth factors, and appropriate scaffolds. Our experiments used IMT as a tissue source, rhBMP-2, rhBMP-4, and rhBMP-7 as cytokines, and ePTFE as a scaffold. Since IMTs are extracted from fetal rats, we recognize that their use is still problematic. However, we showed that IMT differentiates almost equally into bone-like tissue on ePTFE by culturing with rhBMP-2, rhBMP-4, and rhBMP-7 in vitro. Furthermore, this induced bone-like tissue showed ossification potential within one week after implantation and promoted calvarial defect healing over time. Currently, stem cells are mainly used as a cell source for regenerative medicine and tissue engineering because they are pluripotent. IMT may not have pluripotency as stem cells; however, these results suggest that IMT will provide an effective tissue source, and the combination of IMT with rhBMP-2, rhBMP-4, and rhBMP-7 will contribute to bone tissue engineering in the near future.

## Data Availability

The data presented in this study are available on request from the corresponding author. The data are not publicly available due to its confidentiality.
